# The Impact of E-Cigarette Warnings, Warning Themes and Inclusion of Relative Harm Statements on Young Adults’ E-Cigarette Perceptions and Use Intentions

**DOI:** 10.3390/ijerph16020184

**Published:** 2019-01-10

**Authors:** Olivia A. Wackowski, Jennah M. Sontag, David Hammond, Richard J. O’Connor, Pamela A. Ohman-Strickland, Andrew A. Strasser, Andrea C. Villanti, Cristine D. Delnevo

**Affiliations:** 1 Center for Tobacco Studies, Rutgers School of Public Health, New Brunswick, NJ 08901, USA; js2486@sph.rutgers.edu (J.M.S.); delnevo@sph.rutgers.edu (C.D.D.); 2 School of Public Health and Health Systems, University of Waterloo, Waterloo, ON N2L 3G1, Canada; david.hammond@uwaterloo.ca; 3 Department of Health Behavior, Roswell Park Cancer Institute, Buffalo, NY 14263, USA; richard.o’connor@roswellpark.org; 4 Department of Epidemiology & Biostatistics, Rutgers School of Public Health, Piscataway, NJ 08854, USA; ohmanpa@sph.rutgers.edu; 5 Department of Psychiatry, Perelman School of Medicine, University of Pennsylvania, Philadelphia, PA 19104, USA; strasse3@mail.med.upenn.edu; 6 Department of Psychiatry, Vermont Center on Behavior and Health, University of Vermont, Burlington, VT 05405, USA; Andrea.Villanti@uvm.edu

**Keywords:** e-cigarettes, tobacco warnings, risk communication, health communication, risk perceptions

## Abstract

Although e-cigarettes in the United States are required to carry one nicotine addiction warning, little is known about the impact of other potential e-cigarette warning themes, nor about pairing warnings with messages that communicate e-cigarettes’ reduced-harm potential relative to cigarettes. We randomly assigned 876 young adults (ages 18–29) to view e-cigarette ads in a 3 × 2 plus control online experiment that varied by warning theme (i.e., nicotine addiction; nicotine’s impact on adolescent brain development; presence of harmful chemicals) and warning type—i.e., the presence (“relative harm warning”) or absence (“standard warning”) of a relative harm (RH) statement in the warning label (“e-cigarettes may cause harm to health but are less harmful than cigarettes”). Warning believability, informativeness, understandability and support were high across conditions and there were no significant differences by warning theme on e-cigarette harm perceptions or use intentions nor on nicotine (mis)perceptions. Perceived warning effectiveness for discouraging youth initiation was higher for the “brain” and “chemicals” warnings compared to the addiction warning. Warnings with the included RH statement were perceived as less believable and credible and were less frequently correctly recalled. Research should continue to investigate the impact of different e-cigarette warning themes and formats with priority audiences.

## 1. Introduction

Electronic cigarettes (“e-cigarettes”) likely pose substantially fewer health risks to individuals than combustible cigarettes, and might be relevant for use in harm-reduction by smokers, but they are not risk free and their initiation by non-smokers could constitute public health harm [1,2]. Further, research suggests consumers want to know about the potential risks of e-cigarettess [3]. As such, inclusion of warning labels on e-cigarette packaging and advertising is one strategy for informing the public of e-cigarettes’ potential risks, as is done for combustible cigarettes and smokeless tobacco [4]. 

In August 2018, the U.S. Food & Drug Administration (FDA) began requiring that e-cigarette packaging and advertising carry a warning that the products contain nicotine, an addictive chemical. Although some e-cigarette brands previously carried voluntary warnings [5], their contents and format have varied greatly. For example, MarkTen brand ads have included long warnings in small font size measuring around 10% of the ad, while others (e.g., Vuse, Blu) have included messages in small font with low contrast unrecognizable as traditional warnings [6,7]. Warning format is now standardized under new FDA requirements to enhance their noticeability: warnings need to occupy at least 20% of ad space, appear in the upper portion of the ad, include a rectangular border, and use a font size that occupies most of the warning area [8]. They also need to occupy 30% of the front and back of the principal panels of e-cigarette packaging. This is important given that previous research has shown strengthening of warnings to be associated with improvements in warning attention, recall, perceived effectiveness and tobacco risk-related knowledge [9,10].

With respect to warning content, research on the efficacy of nicotine addiction messages has been mixed. One study found that smokers and e-cigarette users exposed to e-cigarette ads with an addiction warning had higher e-cigarette risk beliefs and lower willingness to try e-cigarettes relative to an unexposed group [11]. However, the tested warning stated that “electronic cigarettes are addictive” in contrast to the more indirect wording of the FDA’s warning [“WARNING: This product contains nicotine. Nicotine is an addictive chemical”]. Another study found no impact of e-cigarette warnings, including those similar to the FDA’s, embedded in advertisements on young adult non-smokers’ e-cigarette harm perceptions and use intentions [12]. 

Research suggests that other warning themes might be relevant and potentially more impactful than an addiction warning [6,13], and the FDA could propose additional warnings if appropriate for public health. Some tobacco control experts have noted that e-cigarette warnings could describe negative nicotine effects, such as its harm on adolescent brain development, a message which might resonate more with young people, who are a priority audience for the addiction warning [14]. Indeed, this risk has been highlighted in the 2016 Surgeon General’s Report on e-cigarettes [15] and in the CDC’s public e-cigarette informational materials [16]. Research has also suggested that the presence of harmful chemicals found in e-cigarettes could be a relevant warning theme [6,14,17], with one experimental study finding an effect of ingredient-themed warnings on reducing e-cigarette cravings [17].

On the other hand, the use of strong e-cigarette warnings could have unintended consequences if they contribute to misperceptions that e-cigarettes are equally harmful as cigarettes, and discourage smokers from using them to reduce or quit smoking cigarettes. Nicotine-focused warnings could also potentially contribute to existing nicotine misperceptions (e.g., that nicotine causes cancer) [18,19,20]. Ideally, e-cigarette warnings should inform about risks and discourage initiation by non-smokers and young people without simultaneously increasing misperceptions and discouraging smokers from using them for smoking cessation and harm-reduction [14,21,22]. Following this, research evaluating the effectiveness of e-cigarette warnings should consider their impact on these different audiences.

One potential strategy for minimizing unintended effects could be to pair e-cigarette warnings with a relative harm (RH) statement, ultimately communicating that e-cigarettes may cause harm but are less harmful then combusted cigarettes. Limited research on this topic (mostly on smokeless tobacco) suggests that warning labels that include relative harm statements may improve relative harm perceptions [23,24], but also be viewed as less believable, credible and more ambiguous than standard/traditional warnings [25,26,27,28,29]. Another concern is that the inclusion of relative harm statements might unintentionally encourage use among non-smokers, which would constitute public health harm [24].

Because e-cigarette warnings are a new research area with potential intended and unintended effects, this study had several objectives. First we aimed to examine whether e-cigarette ads with the new larger standardized warnings would have greater impact on young adults’ e-cigarette harm, risk and addiction perceptions, and e-cigarette use intentions compared to e-cigarette ads with no or minimal unstandardized warnings. We also aimed to examine whether exposure to such ads would unintentionally increase misperceptions that e-cigarette and cigarettes are equally harmful, increase nicotine misperceptions, and reduce smokers’ intentions to use e-cigarettes for harm reduction.

Second, we aimed to examine whether warning themes other than nicotine addiction would have a different impact on these same outcomes, and whether other themes would be perceived by young adults to be as effective or more effective than the nicotine addiction warning. 

Finally, we also aimed to explore the potential effects of pairing e-cigarette warnings with a relative harm statement. We hypothesized that those exposed to such warnings would have more accurate e-cigarette/cigarette relative harm perceptions and higher intentions to use e-cigarettes for harm-reduction among smokers. However, we also hypothesized that these warnings would be perceived as less effective and credible, more difficult to understand and recall, and would encourage use among non-smokers. We focused on young adults given their high prevalence of e-cigarette experimentation and priority as a target audience for such warnings. 

## 2. Materials and Methods 

### 2.1. Participants and Procedures

An online experiment was conducted between January–February 2018 with 876 young adults recruited through Amazon’s Mechanical Turk (mTurk; Table 1). Eligibility was limited to registered mTurk participants between the ages of 18–29 located in the United States who had an 80% approval rating from previous mTurk human intelligence tasks (HITs). After a large number of non-smokers initially completed the survey (with approximately 80 non-smokers per condition), we limited eligibility and access to the full survey to current smokers only. Smoking status was determined in the first few questions of the survey, which included distractor questions about other health behaviors (e.g., soda and alcohol consumption, exercise) to mask the focus of the survey. The study was advertised on mTurk as a Health Behavior Survey and the study description did not reveal the focus on e-cigarettes. 

**Figure A1:**
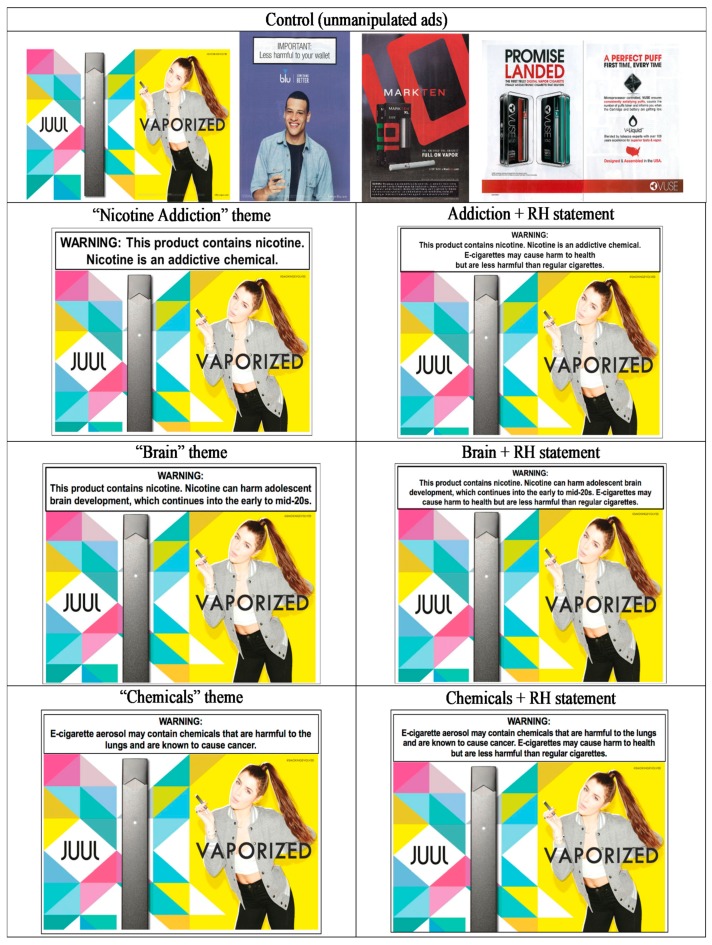
Manipulation of stimuli ads, by condition. NOTE: All four e-cigarette brand ads were viewed in each condition, each containing the same warning, respectively.

Eligible participants were randomly assigned to view e-cigarette ads in one of seven conditions in a 3 (warning theme) by 2 (warning type) between group factorial design with a control group. In the experimental groups, three “warning themes” i.e., (a) nicotine addiction; (b) nicotine’s impact on adolescent brain development; (c) presence of harmful chemicals), were each crossed with “warning type”, in which a relative-harm (RH) statement (“E-cigarettes may cause harm to health but are less harmful than cigarettes”) was either included (“RH warning”) or not included (“standard warning”) in the warning label. Participants in the control group viewed an unaltered set of control ads (see Figure A1). 

Regarding themes, the addiction warning was the FDA’s pending nicotine addiction warning. The “brain” warning stated, “WARNING: This product contains nicotine. Nicotine can harm adolescent brain development, which continues into the early to mid-20s.” The “chemicals” warning stated: “WARNING: E-cigarette aerosol may contain chemicals that are harmful to the lungs and are known to cause cancer.” Message themes were based on formative research [6,14,27] and wording was adapted from the CDC’s e-cigarette fact sheet page at the time of the study [16]. 

Within each experimental group, participants viewed four different e-cigarette ads representing different leading brands (JUUL, Vuse, Blu, MarkTen), which were each manipulated to include the same warning message per their assigned condition. Warnings were sized at approximately 20% of the ad and placed in the upper portion of the ad, per new FDA e-cigarette warning requirements. Ads were presented in random order. Participants in the control groups viewed the same ads in their original un-manipulated form (i.e., as found in the marketplace) (Figure A1, Appendix A). Participants could view each ad for as long as they wished but each ad was displayed for a minimum of seven seconds. After exposure to all four ads, participants completed outcome measures and then viewed a screen with debriefing information corresponding to their condition. The median study completion time was ten minutes and participants were compensated with a $1.75 payment.

### 2.2. Measures

Before viewing the stimuli ads, participants answered questions about their demographics and tobacco use (Table 1). Ever e-cigarette users were defined as those who indicated ever trying an e-cigarette, even one or two times. Current smokers and current e-cigarette users were defined as those indicating they now smoke cigarettes/use e-cigarettes every day or some days, respectively. Immediately after viewing the stimuli, the main dependent variables were measured. Our outcomes of focus were informed by our research objectives and by the Message Impact Framework for tobacco warnings, which suggests that characteristics of warnings, and audiences’ perceptions about warnings may impact whether warnings are noticed and recalled, and may influence warnings’ impact on tobacco product beliefs and use intentions [30]. Question wording for the measures below is available in Supplemental Table S1. 

*E-cigarette Use Intentions.* All respondents were asked about their *likelihood of buying an e-cigarette* in the next 6 months (1 = not at all–5 = extremely likely). We also measured smokers’ *likelihood to use e-cigarettes for harm reduction* by creating an average scale of three items asked of current smokers about their likelihood to use e-cigarettes to cut down on smoking, quit smoking completely, and likelihood of switching to e-cigarettes completely (α = 0.91) [31]. 

*E-cigarette Beliefs.* We assessed *perceived absolute harm of e-cigarettes* by asking respondents how harmful they believed daily e-cigarette use to be to a user’s health (1 = not at all–5 = extremely harmful) [12]. Next, we created a sum score of the *perceived likelihood of disease* (lung cancer, other lung disease, heart disease, oral cancer) (1 = not at all–5 = extremely likely) from daily e-cigarette use over a lifetime (α = 0.92) [32], where higher total scores represented higher perceived risk. *Perceived harm of e-cigarettes compared to cigarettes* was measured by asking how harmful to health daily e-cigarette use is compared to daily cigarette smoking (1 = a lot less harmful–5 = a lot more harmful) [12,33]. We also measured *perceived likelihood of addiction to e-cigarettes* (1 = not at all–5 = extremely likely).

*Nicotine Beliefs.* We measured the extent to which participants agreed that *nicotine causes smoking related cancer* as an indicator of nicotine misperceptions (1 = not at all–4 = very much) and also asked participants to indicate their overall *perceived harmfulness of nicotine to health* (see Table 2) [20]. 

*Warning Recall.* Participants in the experimental groups were asked to type what they could remember of the warning in the ads they viewed in as much detail as possible (unaided recall). Consistent with previous research [34,35], two reviewers independently coded the responses for reference to keywords or concepts that were present in the warnings viewed (Table 3; average Kappa = 0.9). Respondents were then also exposed to the text of all six warnings and asked to select which they recalled viewing on their stimulus ads (aided recall).

*Perceived Warning Effectiveness, Credibility and Support:* Participants in the experimental groups viewed the warning per their condition again and were asked the extent to which they agreed (1 = strongly disagreed–4 = strongly agreed) it was believable, understandable, informative and discouraged e-cigarette use among young people and smokers [36,37,38]. Perceived credibility of the warning was measured adapting Meyer’s credibility scale (5-point semantic differential items: e.g., accurate/inaccurate, biased/unbiased; α = 0.88) [12,39]. Finally participants were asked about the extent to which they agreed (1 = strongly disagreed–4 = strongly agreed) that e-cigarettes should carry warning labels about their potential risks. Results were dichotomized as agreed/disagreed.

*Analysis*: Because of the unbalanced experimental design (3 × 2 plus control), we first tested for mean difference across all seven warning conditions (including control) in the first set of outcomes, and for any interaction effects with smoking status (Model 1, Table 2). We then removed the control group and tested for any effects of warning theme or warning type when the experimental conditions were collapsed across all participants (Model 2, Table 2). For the perceived warning effectiveness outcomes (which were not assessed for the control group), we assessed the effects of warning theme and type, theme x type interactions and interactions with smoking status (Table 4). Any significant interaction effects across all analyses are noted in the results. We used ANOVA tests for semi-continuous outcomes (using Tukey’s post-hoc tests for pairwise comparisons and error-rate control) and Chi-square tests for categorical outcomes (e.g., message recall, warning support). To minimize the likelihood of Type 1 error from multiple testing, we used a more conservative significance level of 0.01 for all analyses. Analyses were completed using SPSS version 25 (SPSS Inc., Chicago, IL, USA).

## 3. Results

*Sample description.* Conditions did not significantly differ by participant demographics, cigarette smoking and e-cigarette use, indicating that randomization was successful. Overall, the sample was evenly split by sex, was predominantly non-Hispanic white (64.3%), and most were employed (75.1%) (Table 1). Participant average age was 25% and 51.1% had at least a college degree.

Over one-third (35.7%) were current smokers and over half of current smokers (54.5%) were current e-cigarette users. About 41% of all participants and 46.0% of smokers had ever noticed a warning on an e-cigarette ad or product package before. 

*E-cigarette use intentions*. There was no significant difference in *likelihood of buying e-cigarettes* across all conditions (see Table 2, Model 1), nor by warning theme or type among the collapsed experimental conditions (Model 2). Among current smokers (n = 313), we also found no significant difference in mean *likelihood of using e-cigarettes for harm reduction* across all conditions (*p* = 0.27) (Table 2, Model 1). However, among the experimental conditions, the main effect for warning type approached significance (*p* = 0.012), with a higher likelihood of using e-cigarettes for harm reduction among smokers exposed to the ads with RH warnings (M = 2.93, SD = 1.1) relative to the standard warnings (M = 2.58, SD = 1.1) (data not in table). 

*E-cigarette Beliefs.* There was no effect of warning condition (nor interaction with smoking status) on perceived absolute harm of e-cigarette use nor on perceived likelihood of disease from e-cigarette use (Table 2). For both outcomes, there were no differences by warning theme or type among the experimental conditions. Perceived harm of e-cigarettes compared to cigarettes was also not significantly associated with warning conditions, nor with warning theme or type when experimental conditions were collapsed (Table 2). 

There was no effect of warning condition (nor interaction with smoking status) on perceived likelihood of addiction (Table 2, Model 1). When experimental conditions were collapsed by theme and type, the main effect of warning theme approached significance (*p* = 0.013, Table 2, Model 2), with scores higher for the FDA addiction warning (M = 3.65, SD = 1.0) relative to the “chemicals” warning (M = 3.38, SD = 0.9, *p* = 0.01), but not relative to the nicotine brain warning (M = 3.54, SD = 1.0). 

*Nicotine Beliefs.* Over half of all participants (56.2%) incorrectly believed nicotine to be the chemical largely responsible for cancer caused by smoking and 54.4% rated nicotine as being very or extremely harmful to health. There was no significant mean differences for either of the nicotine beliefs by warning condition (Table 2) (nor interactions between condition and smoking status), nor by warning theme or type among the experimental conditions. 

*Warning Recall.* Unaided recall of specific warning keywords/concepts was significantly lower in the RH warnings versus the standard versions of the nicotine addiction and brain warning themes (see Table 3). Among those exposed to the standard version of the FDA warning, most recalled the keywords/concepts of “nicotine” (84.1%) and “addiction” (66.7%). For the brain warning (standard version), recall of some reference to age (78%) was higher than for the keywords/concepts “nicotine” (69.3%) and “brain” (65.4%). For the “chemicals” warning, recall of “cancer” was more prevalent than “chemicals” and “lungs” (Table 3). 

When *aided* recall/recognition was measured, there was no significant association between correct recall and warning theme but correct warning recall/recognition was lower among those who viewed the RH warnings (69.5%) versus the standard warnings (84.1%, *p* =< 0.001).

*Perceived warning effectiveness, credibility and support.* Most participants across conditions agreed that the warning they viewed was believable (89.3%), however mean believability was significantly higher for the standard versus the RH warnings (see Table 4). Agreement that the warning was understandable was also high (95.5%) and was not significantly associated with warning type nor theme. Overall, 82.7% of respondents agreed that the warning they viewed would make people more informed about e-cigarette health risks. Mean perceived informativeness did not significantly differ by theme or type (Table 4). 

About 61% agreed that the warning they viewed could discourage young people from starting to use e-cigarettes. Mean agreement was significantly associated with warning type and was higher for the standard (M = 2.88) versus RH warnings (M = 2.45). Agreement was also associated with warning theme, and was lowest for the FDA addiction warning (Table 4). In contrast, fewer respondents overall (37.1%) agreed that the warning they viewed would discourage current smokers from using e-cigarettes, with significant main effects by warning theme and type (Table 4). Smoker discouragement was rated as higher for the standard warnings relative to the RH warnings, and was lowest for the addiction theme relative to the brain and chemicals themes. 

For perceived warning credibility, only the main effect of warning type was significant (F = 23.92, R^2^ = 0.03, *p* < 0.001), with participants rating the standard warnings as more credible (M = 3.88, SD = 1.4) than the RH warnings (M = 3.34, SD = 1.5) (data not in table). 

*Warning Support*. Finally, the vast majority (93.2%) agreed that e-cigarettes should carry warning labels about their potential risks. Support was slightly higher among non-smokers (95.0%) versus current smokers (90.0%) (*p* < 0.01) but did not significantly vary by warning condition.

## 4. Discussion

This study tested the impact of exposure to text warnings on e-cigarette ads in a new standardized format, the impact of different warning themes and the inclusion of a message comparing the harm of e-cigarettes relative to cigarettes. Overall, young adults expressed strong support for e-cigarette warnings and perceived them to be believable, understandable and informative about e-cigarette risks. However, we found few experimental effects of warning exposure on e-cigarette harm and risk perceptions and use intentions. 

Consistent with a previous study with young adult non-smokers [12], we found no significant effect of exposure to the experimental warnings on participants’ e-cigarette harm and addiction beliefs, nor on decreasing e-cigarette use intentions among non-smokers when compared to ads with no or small unstandardized warnings. These null findings may suggest that text-only warnings (even large ones) may have limited impact with young adults when placed in otherwise colorful and interesting e-cigarette advertisements [12]. These findings, along with previous research [8,12,14,34], may suggest that the use of visual elements, such as colored warnings or some relevant pictorials, may be worth exploring given that they might enhance the noticeability, salience and recall of these warnings. 

On the other hand, some of the null findings might be viewed as favorable outcomes. Exposure to the test warnings did not increase misperceptions about nicotine’s role in cancer or harmfulness, nor that e-cigarettes are as harmful as cigarettes. It also did not appear to reduce smokers’ intentions to use e-cigarettes for harm reduction. Together these findings may suggest that the new warning requirements may have minimal unintended consequences in terms of increasing product misperceptions or discouraging use of e-cigarettes for harm reduction. 

Notably, this study also found that most participants perceived the experimental warnings, including the FDA’s addiction warning, to be informative about e-cigarette health risks. This is important given that this is the primary purpose of such warnings [8]. In this study unaided recall of the word “nicotine” was high among those exposed to the FDA’s nicotine addiction warning. This too is important given that research has continued to show that some young people remain unsure of whether the e-cigarettes they use contain nicotine or misperceive their products to be nicotine-free [40,41,42], underscoring a basic need for such clear and conspicuous labeling. In this group of young adults, the other warning themes tested (presence of chemicals and nicotine impact on adolescent brain development) did not significantly outperform the FDA’s addiction warning on any of the main experimental outcome measures including e-cigarette harm/risk perceptions and buying likelihood. Together, these findings support the FDA’s use of the nicotine addiction theme as a starting point for e-cigarette warnings. 

However, the nicotine addiction warning trailed behind the other warning themes in terms of its perceived ability to discourage e-cigarette use among young people. Participants also found the “brain” and “chemicals” warnings to be as believable, informative and credible as the nicotine addiction warning. These findings suggest that these warning themes may deserve additional research attention as potential future warnings, particularly for youth audiences. Indeed, a recent e-cigarette study with adolescents found that while 83% knew that e-cigarettes usually contain nicotine, far fewer knew that e-cigarettes contain harmful chemicals (67%) and might harm teen brain development (49%) [43]. 

This study also explored the impact of including a relative harm statement within the warning label, as has previously been proposed by smokeless tobacco companies and could similarly be proposed for e-cigarettes in the future. Given the calls for increased communications about the relative harms of different tobacco products, it was encouraging to find that these messages were not perceived as being less understandable than the standard warnings, and were not associated with higher e-cigarette use intentions among non-smokers, in contrast to our hypotheses. However, consistent with prior research on this topic [25,26,27,28,29] and our hypotheses, these warnings were perceived as less believable and credible, and also as less likely to discourage e-cigarette use among young people. In line with our expectation, inclusion of the relative harm statements in the warning label also appeared to reduce recall of the risk-related part of the message, potentially because of the increased length of the final overall message and a recency effect of the relative harm information at the end of the message. Future research should continue to examine the effects of various relative harm/modified risk tobacco messages, including appropriate wording, locations and formats that maximize potential public health benefits and minimize unintended consequences. Future research on this topic, as well as on e-cigarette warning labels more generally, should also be examined with more representative samples of participants, as participants in mTurk convenience samples have shown to generally be younger, more educated, have above average cognitive aptitude and be more likely to be white and Asian compared to the general population [44].

## 5. Conclusions

Findings from this study suggest that warning labels with messages about harms beyond nicotine’s addictiveness are perceived as believable, informative, understandable and credible among young adults, may provide novel information, and may discourage e-cigarette use among young people. While inclusion of a relative harm statement in e-cigarette warnings may not unintentionally increase use by non-smokers, it may reduce recall of warning messages in young adults. Further research should continue to identify warning themes, types and execution formats that accurately inform audiences about e-cigarettes.

## Figures and Tables

**Table 1:** Participant demographics and tobacco use (n = 876).

	N	%
**Sex**		
Male	434	49.6
Female	441	50.4
**Race/Ethnicity**		
Non-Hispanic white	563	64.3
Non-Hispanic black	93	10.6
Asian	69	7.9
Hispanic	109	12.5
Other/mixed race	41	4.7
**Education**		
High school of less	115	13.1
At least some college	312	35.6
College degree or more	448	51.1
**Employment**		
Employed for wages	514	58.9
Self-employed	142	16.3
Out of work	69	7.9
Homemaker	32	3.7
Student	116	13.3
**Average Age**		25.0
**E-cigarette Status**		
Ever e-cigarette user	534	61.0
Current e-cigarette user	229	26.2
**Smoking Status**		
Current smoker	313	35.7
Non-smoker	563	64.3

**Table 2:** Impact of Experimental Conditions on E-cigarette Use Intentions, E-cigarette Beliefs and Nicotine Beliefs (n = 876).

	Model 1 (Across All Conditions Including Control) ^a^				Model 2 (Experimental Groups Only) ^b^		
	Main Effects		Interaction		Main Effects		Interaction
	Warning Condition	Smoker Status	Warning Condition x Smoker		Warning Theme	Warning Type	Warning Theme x Type
	F, η^2^, *p*	F, η^2^, *p*	F, η^2^, *p*		F, η^2^, *p*	F, η^2^, *p*	F, η^2^, *p*
Likelihood of buying e-cigarettes ^1^	1.47, 0.010, 0.19	284.46, 0.25, <0.001	2.76, 0.019, 0.012		0.31, 0.001, 0.73	0.99, 0.001, 0.32	1.55, 0.004, 0.21
Likelihood of using e-cigarettes for harm reduction ^1^ (among current smokers only)	1.27, 0.024, 0.27				0.45,0 0.003, 0.64	6.35, 0.024, 0.012	0.06, <0.001, 0.95
Perceived absolute harm of e-cigarettes ^2^	0.45, 0.003, 0.84	38.21, 0.042, <0.001	1.43, 0.010, 0.201		0.093, <0.001, 0.91	1.28, 0.002, 0.26	0.02, 0.000, 0.98
Perceived likelihood of disease from e-cigarette use ^1^	1.09 0.007, 0.37	10.62, 0.012, 0.001	1.05, 0.007, 0.39		0.36, 0.001, 0.70	0.57, 0.001, 0.46	1.41, 0.004, 0.25
Perceived harm of e-cigarettes compared to cigarettes ^3^	0.82, 0.006, 0.55	0.015, <0.001, 0.92	1.36, 0.009, 0.23		0.39, 0.001, 0.68	1.10, 0.001, 0.30	0.94, 0.003, 0.39
Perceived likelihood of addiction to e-cigarettes ^1^	2.17, 0.015, 0.043	10.99, 0.013, 0.001	0.87, 0.006, 0.51		4.39, 0.012, 0.013	0.16, <0.001, 0.69	0.89, 0.002, 0.41
Agreement that nicotine causes smoking related cancer ^4^	0.83, 0.006, 0.55	3.18, 0.004, 0.075	0.36, 0.003, 0.91		1.69, 0.005, 0.19	0.09, <0.001, 0.76	0.18, 0.000, 0.84
Perceived harmfulness of nicotine to health ^2^	0.16, 0.001, 0.99	19.31, 0.022, <0.001	1.06, 0.007, 0.39		0.046, <0.001, 0.96	0.008, <0.001, 0.93	0.30, 0.001, 0.74
	**Mean (Standard Deviation), by condition**						
	Addiction	Addiction + RH	Brain	Brain + RH	Chemicals	Chem. + RH	Control
**E-cigarette Use Intentions**							
Likelihood of buying e-cigarettes ^1^	1.83 (1.1)	1.84 (1.1)	1.77 (1.1)	2.06 (1.2)	1.89 (1.2)	1.83 (1.2)	1.84 (1.0)
Likelihood of using e-cigarettes for harm reduction (among current smokers) ^1^	2.52 (1.2)	2.81 (1.1)	2.61 (1.2)	2.98 (1.1)	2.61 (1.1)	3.0 (1.1)	2.89 (1.3)
**E-cigarette and Nicotine Beliefs**							
Perceived absolute harm of e-cigarettes ^2^	2.29 (1.0)	2.20 (0.9)	2.2 (0.9)	2.26 (0.9)	2.26 (0.9)	2.10 (0.8)	2.13 (0.9)
Perceived likelihood of disease from e-cigarette use ^1^	12.2 (3.9)	12.5 (3.8)	12.1 (3.6)	12.0 (3.9)	12.6 (3.9)	11.8 (3.7)	11.6 (4.1)
Perceived harm of e-cigarettes compared to cigarettes ^3^	2.29 (1.0)	2.20 (0.9)	2.20 (0.9)	2.26 (1.0)	2.26 (0.9)	2.10 (0.8)	2.13 (0.9)
Perceived likelihood of addiction to e-cigarettes ^1^	3.64 (1.1)	3.65 (1.0)	3.5 (1.0)	3.57 (1.0)	3.46 (1.0)	3.30 (1.0)	3.4 (1.0)
Agreement that nicotine causes smoking related cancer ^4^	2.51 (1.0)	2.54 (1.0)	2.68 (1.0)	2.65 (1.0)	2.71 (1.0)	2.63 (1.0)	2.72 (0.9)
Perceived harmfulness of nicotine to health ^2^	3.57 (1.0)	3.63 (1.0)	3.62 (1.1)	3.62 (1.0)	3.66 (1.0)	3.58 (1.1)	3.57 (1.1)

^a^ Model 1 based on Anova analysis for mean differences across all seven warning conditions (including control), with test for interaction with smoking status; ^b^ Model 2 based on two-way Anova analysis for warning theme, warning type and warning theme x type interaction among experimental conditions (control group excluded). F = F statistic η^2^—eta squared, *p* = *p*-value from Anova test. ^1^ (1 = not at all–5 = extremely likely), ^2^ (1 = not at all–5 = extremely harmful), ^3^ (1 = a lot less harmful–5 = a lot more harmful), ^4^ (1 = not at all—4 = very much).

**Table 3:** Unaided recall of warning keywords and concepts by warning theme and type.

% Correctly Recalling Keywords/Concepts for:	Warning Type		*p*-Value *
	Standard	RH Warning	
	%	%	
FDA Nicotine Addiction Warning			
Nicotine	84.1	65.9	0.001
Addiction	66.7	41.1	<0.001
Chemical	43.7	19.4	<0.001
E-cigarettes are less harmful than cigarettes		58.1	
Nicotine Brain Development Warning			
Nicotine	69.3	51.2	0.003
Brain	65.4	49.6	0.011
Age-related	78.0	57.6	0.001
E-cigarettes are less harmful than cigarettes		36.0	
Harmful Chemicals Warning			
Chemicals	36.8	38.7	0.76
Lungs	32.8	21.8	0.051
Cancer	64.0	50.8	0.035
E-cigarettes are less harmful than cigarettes		56.1	

* *p*-values based on Chi-square tests between warning types and recall of each keyword/concept.

**Table 4:** Perceived effectiveness of e-cigarette warnings.

	Focal Statistics for Anova Tests					
	Believable	Understandable	Informative	Discourage Young People	Discourage Smokers	
	F, η^2^, *p*	F, η^2^, *p*	F, η^2^, *p*	F, η^2^, *p*	F, η^2^, *p*	
**Main Effect**						
Warning type	24.32, 0.032, <0.001	5.00, 0.007, 0.026	6.05, 0.008, 0.014	40.44, 0.051, <0.001	20.07, 0.026, <0.001	
Warning theme	3.43, 0.009, 0.033	1.36, 0.004, 0.26	4.14, 0.011, 0.016	13.30, 0.034, <0.001	6.06, 0.016, 0.002	
Smoking status	0.87, 0.001, 0.35	1.47, 0.002, 0.23	0.19, <0.001, 0.66	0.022, <0.001, 0.88	12.07, 0.016, 0.001	
**Interaction Effects**						
Warning type x theme	4.01, 0.011, 0.018	3.58, 0.010, 0.028	0.68, 0.002, 0.508	0.63, 0.002, 0.533	1.08, 0.003, 0.339	
Warning type x smoking status	0, <0.001, 0.989	0.05, <0.001, 0.826	0.16, <0.001, 0.693	0.36, <0.001, 0.549	0.01, <0.001, 0.927	
Warning theme x smoking status	3.11, 0.008, 0.045	3.6, 0.010, 0.028	0.88, 0.002, 0.415	0.19, 0.001, 0.828	0.17, <0.001, 0.844	
Warning type x warning theme x smoking status	2.25, 0.006, 0.106	3.49, 0.009, 0.031	0.5, 0.001, 0.604	0.03, <0.001, 0.975	0.01, <0.001, 0.995	
	**Overall Mean Agreement** **(Across Groups)**	**Mean Agreement** **(St. Deviation) by** **Warning Theme**			**Mean Agreement** **(St. Deviation) by** **Warning Type**	
**Agreed the warning they viewed:**		**FDA**	**Brain**	**Chemicals**	**Standard**	**RH**
Is believable	3.20 (0.7)	3.31 (0.7)	3.17 (0.7)	3.17 (0.8)	3.35 (0.7)	3.10 (0.7)
Easy to understand	3.41 (0.6)	3.45 (0.6)	3.36 (0.6)	3.40 (0.6)	3.46 (0.6)	3.36 (0.6)
Would inform people about e-cigarette risks	3.01 (0.7)	2.92 (0.8)	3.00 (0.7)	3.1 (0.7)	3.07 (0.7)	2.94 (0.7)
Would discourage young people from starting e-cigarettes	2.69 (0.9)	2.47 (0.9) ^b,c^	2.86 (0.8) ^f^	2.73 (0.8) ^f^	2.88 (0.8)	2.45 (0.9)
Would discourage current smokers from using e-cigarettes	2.27 (0.9)	2.12 (0.9) ^b^	2.36 (0.9) ^f^	2.33 (0.9)	2.41 (0.9)	2.12 (0.9)

Note: F = F statistic, η^2^ = partial eta squared, *p* = *p*-value for F statistic. ^f^ = significantly different than FDA nicotine theme, ^b^ = significantly different than Brain theme, ^c^ = significantly different than Chemicals theme, with significance level set at *p* = 0.01.

## Supplementary Materials

The following are available online at http://www.mdpi.com/1660-4601/16/2/184/s1, Table S1: Appendix of Study Measures.
