# Supplementary material for: The Impact of E-Cigarette Warnings, Warning Themes and Inclusion of Relative Harm Statements on Young Adults’ E-Cigarette Perceptions and Use Intentions
Source: Int J Environ Res Public Health. 2019 Jan 10;16(2):184. doi: 10.3390/ijerph16020184 (PMC6352031; doi:10.3390/ijerph16020184)
Supplement: Supplementary file 1 [file ijerph-16-00184-s001.pdf]

Table S1. Appendix of Study Measures.

CIGARETTE AND E-CIGARETTE USE VARIABLES

Do you now smoke cigarettes every day, some days or not at all?

1. Every day
2. Some days
3. Not at all

The next few questions are about "e-cigarettes," including personal vaporizers, vape pens, tanks and mods.

These products are battery powered and include an "e-liquid" that produces vapor or aerosol instead of smoke. Some "e-cigarettes" can be bought as one-time, disposable products, while others can be bought as re-usable kits with cartridges or a tank system.

Some common brands include Vuse, JUUL, MarkTen, Blu, Logic, eGo, and iTaste, but there are many.

Have you ever tried an e-cigarette, even one or two times?

1. Yes
2. No

Do you now use e-cigarettes every day, some days or not at all?

1. Every day
2. Some days
3. Not at all

DEMOGRAPHICS.

The next questions ask about your demographics.

What is your age?

________ years old

Do you currently consider yourself to be:

1. Male
2. Female

Are you Hispanic or Latino, or of Spanish origin?

1. YES
2. NO

Which one or more of the following would you say is your race? Select all that apply to you.

1. White
2. Black or African American
3. Asian
4. Native Hawaiian or other Pacific Islander
5. American Indian, Alaska Native
6. OTHER [SPECIFY ______________________]

What is the highest level of school you completed or the highest degree you received?

1. Grades 1 through 8 (elementary)
2. Some high school
3. High School Graduate or GED
4. Some college or technical school (did not complete or in progress)
5. College degree or Technical School Certification
6. Some graduate or professional school
7. Graduate or professional degree

Which of the following best describe your current status:

1. Employed for wages
2. Self-employed
3. Out of work for more than 1 year
4. Out of work for less than 1 year
5. A homemaker
6. A student
7. Retired

<u>E-CIGARETTE USE INTENTIONS</u>

How likely are you to <u>buy</u> an e-cigarette product in the next 6 months?

1. Not at all likely
2. Not too likely
3. Somewhat likely
4. Very likely
5. Extremely likely

How likely are you to use e-cigarettes as a way to <u>cut down</u> on your cigarette smoking?

*(Ask of those who currently smoke regular cigarettes some days or every day***)**

1. Not at all likely
2. Not too likely
3. Somewhat likely
4. Very likely

5. Extremely likely

How likely are you to use e-cigarettes as a way to quit smoking cigarettes <u>completely</u>?

*(Ask of those who currently smoke regular cigarettes some days or every day***)**

1. Not at all likely
2. Not too likely
3. Somewhat likely
4. Very likely
5. Extremely likely

How likely are you to ever <u>completely switch</u> from cigarettes to e-cigarettes?

*(Ask of those who currently smoke regular cigarettes some days or every day***)**

1. Not at all likely
2. Not too likely
3. Somewhat likely
4. Very likely
5. Extremely likely

<u>E-CIGARETTE BELIEFS</u>

How harmful, if at all, do you think daily use of an <u>e-cigarette</u> is to a user's health?

1. not at all harmful
2. not too harmful
3. somewhat harmful
4. very harmful
5. extremely harmful

How likely or unlikely do you think using an <u>e-cigarette</u> daily would cause an <u>e-cigarette</u> user to develop each of the following diseases in their lifetime?

- **Lung cancer**
  1. Not at all likely
  2. Not too likely
  3. Somewhat likely
  4. Very likely
  5. Extremely likely

- **Other Lung disease (e.g., emphysema)**
  1. Not at all likely
  2. Not too likely
  3. Somewhat likely
  4. Very likely

5. Extremely likely

- **Heart disease**
  1. Not at all likely
  2. Not too likely
  3. Somewhat likely
  4. Very likely
  5. Extremely likely

- **Oral cancer**
  1. Not at all likely
  2. Not too likely
  3. Somewhat likely
  4. Very likely
  5. Extremely likely

Compared with daily cigarette smoking, how harmful to health do you think daily use of e-cigarettes is?

1. a lot less harmful
2. somewhat less harmful
3. about as harmful
4. somewhat more harmful
5. a lot more harmful

How likely is someone to become addicted to e-cigarettes?

1. Not at all likely
2. Not too likely
3. Somewhat likely
4. Very likely
5. Extremely likely

NICOTINE BELIEFS

To what extent, if at all, do you believe the nicotine in cigarettes to be the chemical that causes most of the cancer caused by smoking?

1. Not at all
2. A little
3. Somewhat
4. Very much

How harmful, if at all, do you think nicotine is to health?

1. Not at all harmful
2. Slightly harmful

3. Somewhat harmful
4. Very harmful
5. Extremely harmful

WARNING RECALL

(ask of all except those in CONTROL condition)

The four ads you viewed contained the same warning message. Try to recall what the warning message was, and type what you remember in the box below. Please identify as much detail as possible.

Provide response box:

Which of the following warning statements do you recall seeing on the ads you viewed earlier? (select one)

1. WARNING: This product contains nicotine. Nicotine is an addictive chemical.

2. WARNING: This product contains nicotine. Nicotine is an addictive chemical. E-cigarettes may cause harm to health but are less harmful than regular cigarettes.

3. WARNING: This product contains nicotine. Nicotine can harm adolescent brain development, which continues into the early to mid-20s.

4. WARNING: This product contains nicotine. Nicotine can harm adolescent brain development, which continues into the early to mid-20s. E-cigarettes may cause harm to health but are less harmful than regular cigarettes

5. WARNING: E-cigarette aerosol may contain chemicals that are harmful to the lungs and are known to cause cancer.

6. WARNING: E-cigarette aerosol may contain chemicals that are harmful to the lungs and are known to cause cancer. E-cigarettes may cause harm to health but are less harmful than regular cigarettes

WARNING PERCEPTIONS (perceived effectiveness, credibility, support)

Below is an image of an e-cigarette warning [found on/missing from] the ads you viewed.

[INSERT WARNING Image ON SCREEN that participant viewed]

Please indicate the extent to which you believe this warning:

1. Is believable
2. Is easy to understand
3. Would make people more informed about the health risks of e-cigarettes
4. Would discourage young people from starting to use e-cigarettes
5. Would discourage current cigarette smokers from using e-cigarettes

[response scale: strongly disagree; disagree; agree; strongly agree]

Credibility Scale: Please rate the extent to which you think this warning:

**Can't be trusted**	1	2	3	4	5	**Can be trusted**
**Is inaccurate**	1	2	3	4	5	**Is accurate**
**Is unfair**	1	2	3	4	5	**Is fair**
**Doesn't tell the whole story**	1	2	3	4	5	**Tells the whole story**
**Is biased**	**1**	**2**	**3**	**4**	**5**	**Is unbiased**

Have you ever noticed a warning on an e-cigarette ad or product package before this study?

1. Yes
2. No
3. Don't know/not sure

How much do you agree or disagree that e-cigarettes should carry warning labels about their potential risks?

1. Strongly Agree
2. Agree
3. Disagree
4. Strongly Disagree
